# An Appendiceal Carcinoid Tumor within an Amyand's Hernia Mimicking an Incarcerated Inguinal Hernia

**DOI:** 10.1155/2017/5932657

**Published:** 2017-03-22

**Authors:** Gregorios Christodoulidis, Konstantinos Perivoliotis, Alexandros Diamantis, Dionysios Dimas, Michael Spyridakis, Konstantinos Tepetes

**Affiliations:** ^1^Department of General Surgery, University Hospital of Larissa, Mezourlo, 411 10 Larissa, Greece; ^2^Department of General Surgery, Elpis General Hospital, 115 22 Athens, Greece

## Abstract

*Introduction.* We report the case of an appendiceal carcinoid tumor within an Amyand's hernia, presenting as an incarcerated right inguinal hernia.* Presentation of Case.* A 52-year-old male presented in the emergency department due to a persistent right inguinal pain. Clinical examination revealed a tender right groin mass. Laboratory tests revealed leukocytosis and an increased serum CRP. Under the diagnosis of an incarcerated right inguinal hernia, an emergency operation was taken. Intraoperatively, an inflamed appendix and a part of the cecum were found in the hernia sac. The operation was completed with an appendectomy and a modified Bassini hernia repair. Histological examination revealed a carcinoid tumor, resulting in the performance of a right hemicolectomy.* Discussion.* Amyand's hernia is estimated to account for 0.4% to 0.6% of all inguinal hernias. Coexistence of an Amyand's hernia and a neoplasia is quite rare. Carcinoids are the most frequent tumors found in the appendix, with the size of the primary tumor to be considered the most important prognostic factor and the basis upon which the operative plan is decided.* Conclusion.* A malignancy of the appendix should always be in the differential diagnosis of a right inguinal mass, in order to provide optimum surgical treatment.

## 1. Introduction

Amyand's hernia is defined as the presence of a vermiform appendix in an inguinal hernia sac. It is named on behalf of Claudius Amyand, a French sergeant surgeon, who reported the treatment of a perforated appendix in the inguinal hernia sac of an eleven-year-old boy [1]. According to a recent review [2], the incidence of Amyand's hernia is currently estimated between 0.4% and 0.6%, with the rate of occurrence in the pediatric population being threefold higher. Inflammation of the herniated appendix has a prevalence of about 0.1%.

Carcinoid tumors, a form of slow-growing neuroendocrine neoplasms, represent 0.66% of all malignancies [3]. Coexistence of an appendiceal neoplasm and an Amyand's hernia has been rarely described [4, 5], while the presence of a carcinoid tumor of the appendix within an inguinal hernia sac is an even rarer entity [6].

Therefore we report the case of an Amyand's hernia, presenting in our tertiary surgical unit as an incarcerated right inguinal hernia, where, due to intraoperative findings, an appendectomy was performed. Histological examination of the specimen revealed the presence of a carcinoid tumor, resulting in the performance of a right hemicolectomy.

This case is reported according to the SCARE guidelines [7].

## 2. Presentation of Case

A 52-year-old male presented in the emergency department of our hospital due to a persistent abdominal pain, located in the right inguinal area. The aggravation of the symptoms, along with the onset of fever and retention of gases and stools for 18 hours, forced him to seek medical advice. Regarding the past medical history of the patient, he was under medical treatment for hypertension and was not submitted, previously, to any kind of operation.

Upon arrival, the vital signs of the patient were temperature of 37.7°C, blood pressure of 135/68 mmHg, heart rate of 89 bpm, and respiratory rate of 18/min. Clinical examination revealed a right groin mass, with localized tenderness, during palpation. During auscultation, high pitched bowel sounds were found. No remarkable findings were discovered in digital rectal examination.

As far as laboratory tests were concerned, leukocytosis (15,800/*μ*L, neutrophils 89%) and an increased value of serum CRP (2 mg/dL, normal values ≤ 0.7 mg/dL) were found. All the other laboratory tests (e.g., haematocrit, platelets count, electrolytes, urea, creatinine, SGOT, and SGPT) were within normal values. An abdominal X-ray revealed a few small intestine gas-fluid levels.

Initial resuscitation included the placement of a nasogastric tube and a Foley catheter and the intravenous administration of fluids and broad-spectrum antibiotics. Based on the above-mentioned findings, the preoperative diagnosis of obstructive ileus due to an incarcerated right inguinal hernia was made. With the patient's consent, the decision for an emergency operation was taken.

**Figure 1:**
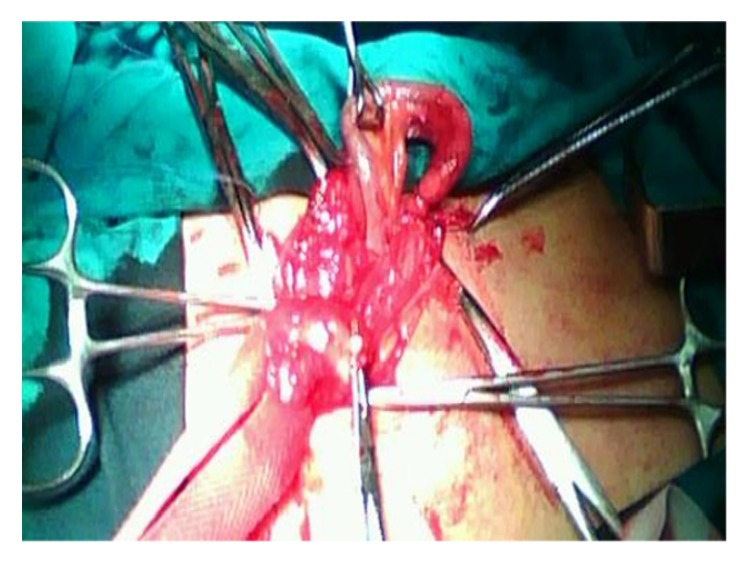
Intraoperative finding of an Amyand's hernia.

After a right inguinal incision was made, further dissection through the anatomical planes revealed an indirect right inguinal hernia. Inside the hernia sac, an inflamed appendix and a part of the cecum were found (Figure 1). Applying gentle manipulation, the rest of the cecum was pulled through the internal inguinal ring and then the bowel was assessed for possible ischemia. Given the confirmed bowel viability and the inflammation of the appendix, an appendectomy was performed, the stump was inverted, and the cecum was returned to the peritoneal cavity. The operation was completed with the hernia repair, through a modified Bassini technique. The decision for not placing a mesh was based on the possible contamination due to the inflamed appendix.

**Figure 2:**
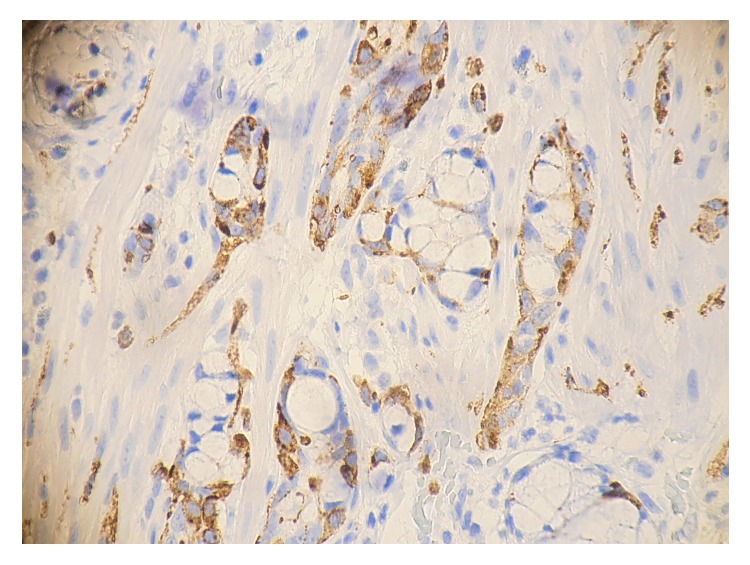
Goblet cell carcinoid of the appendix. The tumor cells show positivity of synaptophysin.

**Figure 3:**
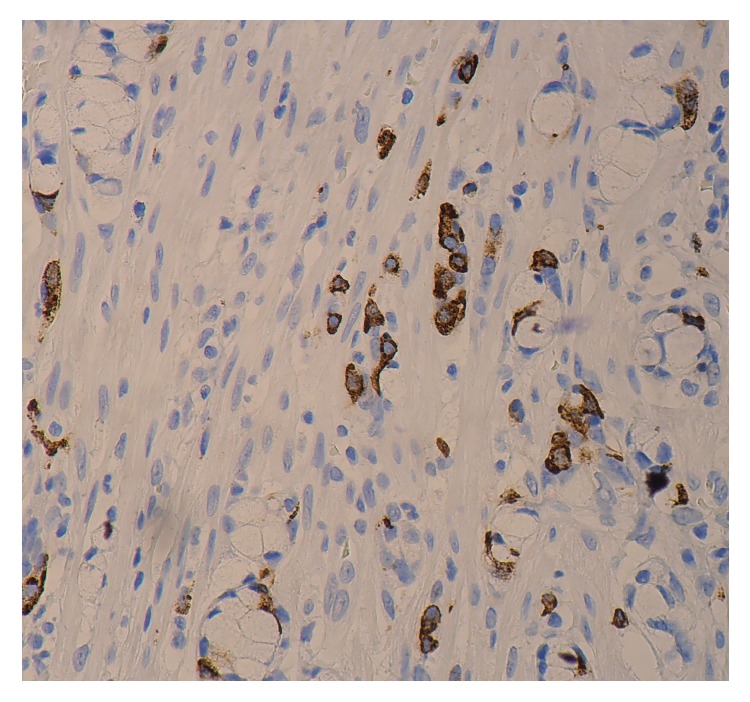
Goblet cell carcinoid of the appendix. Immunohistochemically, the endocrine cell component is positive for chromogranin.

The postoperative period was uneventful, with the patient returning to oral diet the next day and being discharged from the hospital the day after, with instructions for antibiotic treatment and reevaluation upon the histopathological report. A few days later, histopathology of the specimen revealed a 2.2 cm goblet cell carcinoid tumor located in the appendix tip. Both the serosa and the mesoappendix were not infiltrated and a R0 excision was confirmed. Immunohistochemically, the tumor cells were positive for synaptophysin and chromogranin (Figures 2 and 3).

As a result, the patient underwent a full oncologic workup, including measurement of 5-hydroxyindoleacetic acid (5-HIAA) and chromogranin A (CgA) baseline levels and a chest and abdomen CT scan. Moreover an octreoscan was performed, which was not positive for any active distal metastases. The patient was then, subsequently, submitted to a right hemicolectomy. The postoperative period was uneventful, with the patient returning to oral diet a few days later and being discharged from the hospital without any complication. During the first year of follow-up the patient remained asymptomatic and both the levels of biomarkers and scintigraphy were negative, with no sign of disease recurrence.

## 3. Discussion

 Amyand (1660–1740), a sergeant surgeon at St George's Hospital, on December 6, 1735, operated on an 11-year-old boy with an inflamed appendix, inside a right inguinal hernia sac. Due to the chronicity of the inflammation, the patient had developed a fistula and was finally submitted to an appendectomy [8]. As a result of this first report of this rare condition, Amyand's hernia was named after the homonymous renowned surgeon [1]. In a recent literature review by Michalinos et al. [2], Amyand's hernia is estimated to account for 0.4% to 0.6% of all inguinal hernias. Moreover, the rate of inflammation of the herniated appendix is 0.1%. Children have a three times higher probability of presenting with an Amyand's hernia than adults [9], while the prevalence is higher in males. Age distribution of Amyand's hernia peaks in neonates and elderly people [10, 11].

Clinical manifestation of an Amyand's hernia is usually that of a right inguinal mass, resembling an inguinal hernia [2]. Symptoms like pain, right groin tenderness, fever, or even vomiting may also appear, depending on the inflammatory status of the appendix [2]. Due to these, an Amyand's hernia is easily confounded with a strangulated inguinal hernia and, as a result, diagnosis is usually made during the operation [2]. Modalities, such as ultrasound (US) and computed tomography (CT) scans, proved to be valuable in the diagnosis of Amyand's hernia, as they can identify the presence of a tubular structure, connecting with the cecum, inside the inguinal canal [4, 12].

 Losanoff and Basson [13] proposed a classification system and a treatment guideline for Amyand's hernias. More specifically, in type I hernia, there is no sign of inflammation, the appendix is normal, and a mesh hernioplasty is recommended. Furthermore, in type II Amyand's hernia, the inflammation of the appendix is localized inside the hernia sac, and, therefore, the operation of choice is appendectomy and endogenous repair of the hernia due to a possible surgical site infection after a mesh placement. In the next grade, that is, type III, due to the presence of peritonitis laparotomy, appendectomy and a primary hernia repair are required. Finally, in type IV, the inflammatory process in the appendix is associated with an underlying abdominal pathology. In this scenario, treatment of the Amyand's hernia includes both appendectomy and hernia repair and also an appropriate diagnostic and operative sequence of the underlying pathology.

Coexistence of an Amyand's hernia and a neoplasia is quite rare [2]. Salemis et al. [14] reported an appendiceal villous adenoma, while there are also only a few cases of cystadenomas of the appendix [15, 16]. Moreover, appendiceal adenocarcinomas within an Amyand's hernia are rarely observed [4, 5]. There is currently only one known report [6] of a carcinoid tumor inside an inguinal hernia validating, thus, the scarcity of the above-mentioned pathology.

According to the Surveillance, Epidemiology, and End Results program [17], the incidence rate of carcinoid tumors is 2.47 and 2.58 per 100,000 per year for men and women, accordingly. Moreover, a slight increase in the incidence rate is observed in black people. From a series of 20,436 patients, it was found that 0.66% of all neoplasias were carcinoid tumors [18]. Gastrointestinal system is the most frequent site where carcinoid tumors arise. The appendix has an increased rate of carcinoid occurrence, since 17% of all gastrointestinal carcinoids are located in this part of the GI tract [3]. In fact, carcinoids are the most frequent tumors found in the appendix. Regarding the growing location of a carcinoid tumor, the distal third of the vermiform appendix is the commonest site [3]. Although laboratory tests, such as the level of 5-HIAA in a 24-hour urine sample and serum analysis of CgA, are useful in the diagnosis of a carcinoid tumor, final confirmation is provided by histology and positive immunohistochemical staining for chromogranin A or synaptophysin [3]. The size of the primary tumor is considered, among others, the most important prognostic factor and the basis upon which the operative plan is decided, since patients with tumors >2 cm should be submitted to a right hemicolectomy [3, 19]. The prognosis of appendiceal carcinoid tumors is in favor of tumors <2 cm given their lower potential for distal metastases. In local disease the overall 5-year survival rate is estimated at about 94% and in case of distal metastases it is diminished at the level of 34% [3].

## 4. Conclusion

An appendiceal carcinoid tumor within an Amyand's hernia is a rarity. Clinical manifestations often mimic those of an incarcerated right inguinal hernia, rendering the correct preoperative diagnosis very difficult. Therefore, a malignancy of the appendix should always be in the differential diagnosis of a right inguinal mass, in order to provide optimum surgical treatment.

## Appendix

See Figures 1, 2, and 3.
